# Effects of *Dioscorea oppositifolia* L. on growth performance, biochemical indicators, immunity, and intestinal health of weaned piglets

**DOI:** 10.3389/fvets.2025.1529881

**Published:** 2025-04-25

**Authors:** Xiang-Yi Pan, Zheng-Ying Qiu, Chen Liu, Chuan Wang, Xiaowu Wang, Li-Na Huang, Si-Fan Li, Xiong-Wei Shi, Shao-Guang Ge, Rui-Hua Xin

**Affiliations:** ^1^Lanzhou Institute of Husbandry and Pharmaceutical Sciences, Chinese Academy of Agricultural Sciences (CAAS), Lanzhou, China; ^2^Engineering and Technology Research Center of Traditional Chinese Veterinary Medicine of Gansu Province, Lanzhou, China; ^3^Key Laboratory of Veterinary Pharmaceutical Development of Ministry of Agriculture and Rural Affairs of P.R. Lanzhou, Lanzhou, China; ^4^Sichuan SDIC Qiangshan Technology Group Co., Ltd., Mianyang, China; ^5^School of Pharmacy, State Key Laboratory of Applied Organic Chemistry, Lanzhou University, Lanzhou, China

**Keywords:** *Dioscorea oppositifolia* L., weaned piglets, growth performance, immune performance, intestinal health, gut microbiome

## Abstract

**Introduction:**

Weaning stress syndrome in piglets seriously endangers the healthy development of the breeding industry. *Dioscorea oppositifolia* L. (Chinese yam, YAM) has activities such as boosting immunity and regulating gastrointestinal function. In order to explore the potential efficacy of YAM on weaned piglets, this study aimed to investigate the effects of growth performance, immune function, intestinal health and intestinal flora composition of weaned piglets.

**Methods:**

Forty-eight 28-day-old weaned piglets were randomly divided into a control group, YAML group and YAMH group, with 0, 1 and 2% YAM added to the basal diet, respectively. During the experiment, the piglets’ feed intake was recorded, and blood and fecal samples were collected. After the feeding period, intestinal tissue samples and colon content samples were collected for testing.

**Results:**

The results showed that adding YAM to the diet can lower the incidence of diarrhea in weaned piglets, improve growth performance and nutrient digestibility, and reduce serum enzyme activity alanine aminotransferase (ALT), total cholesterol (TC) and low-density lipoprotein cholesterol (LDL-C); in addition, YAM can also increase serum immunoglobulins (Ig) and antibody titers, regulate the level of inflammatory factors, and promote the expression of secretory immunoglobulin A (SIgA) protein in the intestine. Furthermore, supplementation with YAM can increase the villus height (VH), the ratio of villus height to crypt depth (V/C), and the expression of Tight junctions (TJs), and also has a positive regulatory effect on the intestinal flora.

**Discussion:**

In summary, YAM alleviates weaning stress syndrome in piglets by promoting growth performance, improving immune function and disease resistance, improving intestinal morphology and mucosal immunity, and regulating the intestinal microbial composition of piglets. This provides a theoretical basis for the development and application of YAM as a new plant-derived feed additive.

## 1 Introduction

With the development and expansion of pig farming, early weaning of piglets has become a common practice to improve productivity (1, 2). Typically, piglets are weaned at 3–4 weeks of age. During weaning, piglets experience separation from the sow and transition from breast milk to solid feed, requiring them to adjust to new nutritional and environmental conditions. Coupled with immature immune systems and underdeveloped digestive functions, this transition makes piglets highly prone to weaning stress syndrome (3, 4). Weaning stress leads to increased inflammatory factors in the body, activates the immune system, alters intestinal morphology and function, impairs intestinal barrier integrity, and disrupts the gut microbiota (5, 6). These changes contribute to stunted growth, increased susceptibility to pathogens, diarrhea, and, in severe cases, mortality in piglets (7). Thus, finding ways to alleviate weaning stress, improve immunity, promote intestinal health, and in animal nutrition (8).

In order to avoid the harm to farming and human health caused by over-reliance on antibiotics, substances such as probiotics (9), organic acids (10) and plant extracts are now often used in animal husbandry to alleviate the problem of weaning piglet diarrhea (11, 12). Feed additives of herbal origin are attracting increasing attention in animal husbandry because they are rich in polysaccharides and flavonoids, which have potential value in regulating immunity, oxidative activity and maintaining intestinal health. Studies have shown that soy isoflavones can improve the muscle quality, nutritional and flavour qualities and antioxidant capacity of grass carp (13); dandelion flavonoids can enhance the digestion, immunity and antioxidant capacity of fish (14, 15). In addition, adding thyme to pig feed can increase the number of white blood cells, immunoglobulin (Ig) A and IgG levels in pig plasma (16), while adding *Echinacea purpurea* L. can promote lymphocyte proliferation, increase antibody production, and increase the protein and immunoglobulin content of sow colostrum (17).

*Dioscorea oppositifolia* L. (Chinese yam, YAM) is the dried rhizome of *Dioscorea oppositifolia* Thunb, a plant of the genus *Dioscorea* in the family *Dioscoreaceae*, which is mainly distributed in China, India, Japan, Korea, and other Asian regions (18). YAM is rich in active ingredients, including polysaccharides, mucins, and phenols (19), which contribute to its various nutritional and medicinal benefits (20, 21). Recent studies indicate that YAM may also offer potential advantages in livestock and poultry farming. For instance, dietary supplementation with YAM polysaccharides has been shown to regulate intramuscular fat and fatty acid content, as well as to promote muscle tissue development in broilers (22). Additionally, YAM polysaccharides-copper complexes have been reported to improve growth performance, immunity, and antioxidant capacity of broilers (23). Studies have shown that YAM starch is high digestible in the ileum of pigs and promotes short-chain fatty acid (SCFA) production, enhancing digestive and absorptive functions and improving growth performance in weaned piglets (24, 25). However, the effects of YAM on the immune function of weaned piglets remained unexplored.

This study aimed to investigate the alleviating effect of YAM supplements on the growth performance of weaned piglets. Key parameters, including body weight gain, feed conversion ratio, diarrhea incidence, serum biochemical indicators, immune function, intestinal barrier integrity and intestinal flora composition, were systematically evaluated to provide a theoretical basis for the development of YAM as a new green feed additive.

## 2 Materials and methods

### 2.1 Animal care

All animal studies and related protocols in this work were approved by the Animal Ethics Committee of Lanzhou Institute of Husbandry and Pharmaceutical Sciences of CAAS (Licence No. SCXK Gansu 2023–021).

### 2.2 Experimental design and animal management

Forty-eight PIC crossbred piglets (producted in September 2023) with similar body weights (11.39 ± 0.07 kg) provided by Sichuan Guotou Qiangshan Science and Technology Group Co., were randomly divided into three groups of 16 piglets each and kept in a single pen. The control group (control) received the basal diet, the low-dose group (YAML) received the basal diet supplemented with 1% YAM, and the high-dose group (YAMH) received the basal diet supplemented with 2% YAM. YAM was obtained from Zhengde Tang Pharmaceutical Co., Ltd., dried, crushed, and passed through a 25-mesh sieve, it is dispensed and stored in a cool, dry place until use. The experiment lasted for 28 days with ad libitum drinking and feeding ambient temperature maintained at 28°C ± 2°C and relative humidity of 65% ± 5%. The composition of the basal diet is shown in Table 1. The primary nutrients in YAM were analyzed according to the GB/T6434-2022, GB/T6437-2018, GB/T6436-2018, GB/T6438-2007, GB/T6433-2006, GB/T20806-2022, GB/T6435-201, GB/T6432-2018 standards. The results indicated that YAM contained 90.92% neutral detergent fiber, 4.6% crude fiber, 1.7% crude ash, 0.6% crude fat, 0.243% total calcium, 0.11% total phosphorus, 7.44% moisture, and 8.55% crude protein, respectively.

**Table 1:** Composition and nutrient levels of the basal diets (air-dry basis, %).

Ingredients	Content	Nutrient level^b^	Content
Corn	28.72	Digestion energy(Kcal/Kg)	3446.24
Broken rice	20.00	Crude protein	17.00
Soybean-puffed	8.00	Crude fiber	2.58
Soybean meal	14.88	Calcium	0.69
Glucose	2.00	Total phosphorus	0.49
Fish meal	2.00	Total lysine	1.33
Soy sauce	0.39	Total methionine	0.44
White sugar	2.0	Total threonine	0.86
Whey powder	3.0	Total Sulfur Amino acids	0.72
Whole milk treasure	5.0	Total Tryptophan	0.19
L-lysine hydrochloride	0.35	Total Isoleucine	0.70
Methionine	0.15	Total Valine	0.80
L-threonine	0.23		
High-temperature resistant phytase	0.03		
Complex enzymes	0.05		
Kejian peptide	0.20		
Wheat	10.00		
3% nursery feed premix^a^	3.00		
Total	100		

^aPremix provided per kilogram of basal diet: 9555 IU vitamin A, 3000 IU vitamin D3, 81 IU vitamin E, 3 mg vitamin K3, 3 mg vitamin B, 10.2 mg vitamin B2, 6 mg vitamin B6, 0.036 mg vitamin B12, 0.24 mg D-biotin, 30 mg D-pantothenic acid, 36 mg niacinamide, 300.06 mg choline chloride, 135 mg Fe, 115 mg Cu, 60 mg Mn, 1144.5 mg Zn, 118.2 mg methionine, 1,638 mg L-lysine.^

^bNutrient levels were calculated values.^

### 2.3 Sample collection

Blood samples were collected from the anterior vena cava of 10 selected piglets per group on days 0, 14, and 28 for subsequent analysis. At the end of the 28-day experiment, following a 12-h fasting period, the piglets were euthanized via intravenous injection of sodium pentobarbital. Samples from the duodenum, jejunum, ileum, and colon contents were then collected and stored at-80°C for subsequent testing. Fecal samples were individually collected from each piglet for 4 consecutive days on days 25, 26, 27, and 28 days of the experiment. The collected feces were combined, mixed, and stored at-20°C. Fecal samples from the final 4 days of the experiment were dried and ground into powder, and a portion of the feed was retained. The crude protein (CP) and crude fat (EE) content in the diet and fecal samples were determined following the methods outlined in GB/T 6432–2018 and GB/T 6433–2006 standards.

### 2.4 Growth performance and diarrhea rate

The body weight of each piglet was measured on days 0 and 28 of the experiment. The daily feed intake of each group of piglets was recorded throughout the experiment to calculate the average daily gain (ADG), average daily feed intake (ADFI) and feed-to-gain ratio (F/G). Fecal quality was scored every morning from day 0 to day 28 by two independent observers, using a 4-point scoring system: 0 = normally shaped feces, 1 = soft feces, 2 = mildly fluid feces, and 3 = severely watery and frothy feces, based on a previously established method (17). Piglets with an average fecal score > 1 were classified as having diarrhea. The diarrhea rate was calculated according to the following formula:

Diarrhea rate (%) = [(the number of diarrhea pigs×diarrhea days) /(total number of pigs×experimental days)] × 100.

### 2.5 Serum biochemical analyses

The activities of albumin (ALB), alanine aminotransferase (ALT), alkaline phosphatase (ALP), glucose (GLU), total cholesterol (TC), triglyceride (TG), high-density lipoprotein cholesterol (HDL-C), low-density lipoprotein cholesterol (LDL-C), and blood urea nitrogen (UREA) were determined using a fully automatic biochemistry analyzer (BS-240VET; Shenzhen, China).

### 2.6 ELISA

The concentrations of IgA (Wavelength: 450 nm, No. JL13829), IgG (Wavelength: 450 nm, No. JL13296), IgM (Wavelength: 450 nm, No. JL11610), porcine pseudorabies virus antibody (PRV-Ab)(Wavelength: 450 nm, No. JL49000), and classical swine fever virus antibody (CSFV-Ab) (Wavelength: 450 nm, No. JL47166) in serum were determined on day 0, 14 and 28, strictly according to the instructions of ELISA kit. Additionally, serum levels of tumor necrosis factor-*α* (TNF- α) (Wavelength: 450 nm, No. JL13203), interleukin-1β (IL-1β) (Wavelength: 450 nm, No. JL21874), interferon-*γ* (IFN-γ) (Wavelength: 450 nm, No. JL11792), Interleukin-6 (IL-6) (Wavelength:450 nm, No. JL21880) and interleukin-10 (IL-10) (Wavelength: 450 nm, No. JL21866) were measured on day 28. The above reagents were purchased from Shanghai Jianglai Technology Co.

### 2.7 Immunohistochemical staining

The duodenum, jejunum, and ileum were fixed in 4% paraformaldehyde, dehydrated, paraffin-embedded, and sectioned into 5 μm-thick slices. These slices were stained with hematoxylin and eosin (H & E) to observe the morphology of intestinal tissues under a scientific microscope (DM 4000B, Leica, Germany). The villus height (VH), crypt depth (CD), and the ratio of villus height to crypt depth (V/H) were measured using Image-pro plus 6.0 software. For immunohistochemical analysis, tissue sections were dewaxed, rehydrated, and subjected to antigen retrieval. The sections were then treated with 3% hydrogen peroxide and blocked with serum. The primary antibody was incubated overnight at 4°C, followed by incubation with the secondary antibody at room temperature. Color development was facilitated, and nuclear staining was performed using hematoxylin. The expression of small intestinal secreted immunoglobulin A (SIgA) was quantified using Image J software.

### 2.8 Western blotting analysis

Protein extract preparation and protein blotting were performed as in our previous study (26). Protein samples were separated by 10% SDS-PAGE gel and primary antibody was incubated overnight. The secondary antibody was incubated at room temperature for 1 h, followed by dropwise addition of ECL solution on nitrocellulose membrane, detection using Biosciences Imager and data analysis using ImageJ software. The gray value of the target proteins was analyzed with ImageJ software, with *β*-actin (1: 1000) serving as the internal reference. Protein expression levels of Claudin-1 (1: 1000), ZO-1 (1:1000), Occludin-1 (1: 1000), SIgA (1: 1000), β-actin (1: 1000), were quantified, and statistical analysis was performed.

### 2.9 Quantitative real-time PCR analysis

Gene expression was consistent with previous studies (26). Total RNA was extracted with TRIzol reagent, and the concentration of RNA was detected by an ultra-micro spectrophotometer, and then reverse transcribed into cDNA using the EVO reverse transcription kit. RT-qPCR was performed using the Applied Biosystems Real-Time Fluorescence Quantitative PCR System and SYBR Green qPCR premix. qPCR was performed using the Applied Biosystems Real-Time Fluorescence Quantitative PCR System and SYBR Green qPCR premix. qPCR included SYBR qPCR mix, forward and reverse primers, cDNA, and DEPC-treated water. Specific primers are listed in Table 2.

**Table 2:** Primer sequences.

Gene	Forward primer	Accession number	Tm	Product size (bp)
*Occludin-1*	F:5’-CAGGTGCAAAATCAGATTG-3′	NM-001163647.2	60.68	167
	R:5′- ATGTCGTTGCTGGGTGCATA-3′		60.04	
*Claudin-1*	F:5′- ATGACCCCAGTCAATGCCAG-3′	NM-001244539.1	60.03	443
	R:5′- CCCTCTCCCCACATTCGAGA-3′		60.69	
*ZO-1*	F:5′- CAGGTGCAAAATCAGCAAC-3′	NM-003356947.4	55.60	357
	R:5′- TCACAGTGTGGTAAGCGCAG-3′		60.60	
*β-actin*	F:5′ -GGTCACCAGGGCTGCTTT-3’	NM-003357928.4	59.69	216
	R:5′ -ACTGTGCCGTTGACCTTGC-3’		59.75	

### 2.10 16S rRNA colony analysis

Total genomic DNA was extracted from twelve samples using the TGuide S96 Magnetic Soil /Stool DNA Kit (Tiangen Biotech Beijing Co., Ltd.), following the manufacturer’s instructions. The hypervariable region V3-V4 of the bacterial 16S rRNA gene was amplified using the primer pairs 338F: 5′- ACTCCTACGGGAGGCAGCA-3′ and 806R: 5′- GGACTACHVGGGTWTCTAAT-3′. PCR products were analyzed by agarose gel electrophoresis and purified using the Omega DNA purification kit (Omega Inc., Norcross, GA, USA). The purified PCR products were then collected, and paired-end sequencing was performed on the Illumina Novaseq 6,000 platform. Alpha diversity (Simpson index) at the operational taxonomic unit (OTU) level was measured, and beta diversity analyses were performed to assess structural variation in the colonic microbial community. These analyses were performed using USEARCH (version 10) and QIIME2 (2020.6) software.

### 2.11 Data analysis

Data were plotted using GraphPad Prism 9 software and analyzed using IBM SPSS 27. Statistical analyses included One-way ANOVA and independent-samples t-test. Results were expressed as mean ± SEM, with significance considered at *p* < 0.05.

## 3 Results

### 3.1 Effect of YAM on growth performance and serum biochemical of weaned piglets

As shown in Table 3, the inclusion of YAM in the piglet diet resulted in an increase in final BW and ADG, along with a decrease in F/G ratio and Diarrhea rate in the YAM group. A significant difference was observed between the YAMH group and the control group (*p* < 0.05). Additionally, YAM supplementation significantly improve the digestibility of EE and CP in the experimental group (*p* < 0.05). As presented in Table 4, dietary supplementation with YAM for 28 days did not significantly effect the serum levels of ALB, ALP, GLU, UREA, TG and HDL-C in weaned piglets (*p* > 0.05). Howerer, the serum levels of ALT, TC and LDL-C were significantly reduced in the YAMH group (*p* < 0.05).

**Table 3:** Effect of YAM on growth performance of weaned piglets.

Item	Control	YAML	YAMH	*p* value
Initial BW (kg)	11.21 ± 0.11	11.46 ± 0.13	11.51 ± 0.12	0.839
Final BW (kg)	18.00 ± 0.47^b^	18.90 ± 0.60^ab^	20.24 ± 0.62^a^	0.02
ADFI (kg/d)	0.63 ± 0.01	0.60 ± 0.01	0.64 ± 0.01	0.061
ADG (Kg/d)	0.24 ± 0.02^b^	0.27 ± 0.02 ^ab^	0.32 ± 0.02^a^	0.449
F/G	2.78 ± 0.24^a^	2.45 ± 0.23 ^ab^	2.11 ± 0.14^b^	0.095
Diarrhea rate (%)	4.98 ± 0.64^a^	3.62 ± 0.39 ^b^	2.39 ± 0.21^b^	0.001
EE	89.54 ± 1.10^b^	93.57 ± 0.78^a^	95.06 ± 0.77^a^	0.005
CP	93.50 ± 0.48^b^	95.28 ± 0.31^a^	94.88 ± 0.29^a^	0.019

Values are represented as the mean and SEM. ^a–b^ Means within a row without a common superscript differ significantly (*p* < 0.05). In this study,piglets in the control group were fed a basal diet, while the YAML group received the basal diet supplemented with 1%YAM, and the YAMH group received the basal diet supplemented with 2%YAM, the same below. The key parameters measured included BW, body weight; ADFI, average daily feed intake; ADG, average daily gain; F/G, feed to gain ratio; CP, crude protein, EE, crude fat.

**Table 4:** Effect of YAM on serum biochemical of weaned piglets.

Item	Control	YAML	YAMH	*p* value
ALB (g/L)	22.15 ± 0.50	22.75 ± 0.74	22.37 ± 0.88	0.840
ALP (U/L)	200.00 ± 27.57	211 ± 18.12	217.93 ± 24.42	0.864
ALT (U/T)	64.23 ± 5.36^a^	51.87 ± 3.49^b^	43.09 ± 2.77^b^	0.004
GLU (mmol/l)	4.61 ± 0.23	5.14 ± 0.17	4.83 ± 0.30	0.301
UREA (mmol/l)	3.69 ± 0.21	3.17 ± 0.27	3.61 ± 0.13	0.195
TC (mmol/l)	2.30 ± 0.10^a^	2.12 ± 0.12^ab^	1.97 ± 0.05^b^	0.066
TG (mmol/l)	0.53 ± 0.05	0.47 ± 0.04	0.49 ± 0.02	0.482
HDL-C (mmol/L)	0.61 ± 0.04	0.63 ± 0.04	0.63 ± 0.04	0.928
LDL-C (mmol/L)	1.39 ± 0.04^a^	1.24 ± 0.11^ab^	1.12 ± 0.06^b^	0.063

Values are represented as the mean and SEM. ^a–b^ Means within a row without a common superscript differ significantly (*p* < 0.05). Various serum biochemical parameters were measured, including ALB, albumin; ALT, alanine aminotransferase; ALP, serum alkaline phosphatase; GLU, glucose; TC, total cholesterol; TG, triglyceride; HDL-C, high-density lipoprotein cholesterol; LDL-C, low-density lipoprotein cholesterol; UREA, blood urea nitrogen.

### 3.2 Effect of YAM on immune-related indices in serum of weaned piglets

The serum immunity indexes are shown in Table 5. The results indicated that the levels of PRV-Ab and CSFV-Ab in weaned piglets were significantly increased (*p* < 0.05) on the 14th day after the addition of YAM. Similarly, serum Ig levels, including IgA, IgG and IgM, were significantly higher (*p* < 0.05) in the YAMH group on day 14. Meanwhile, YAM supplementation in the diet reduced the serum levels of pro-inflammatory factors IL-6, TNF-*α* and IL-1β, while it increased the levels of IL-10 and IFN-*γ* in weaned piglets.

**Table 5:** Effect of YAM on immune-related indices in serum of weaned piglets

Item	Control	YAML	YAMH	*p* value
PRV-Ab (ng/ml)				
0d	3.65 ± 0.11	3.39 ± 0.13	3.57 ± 0.11	0.304
14d	3.67 ± 0.34^b^	4.39 ± 0.18^ab^	4.58 ± 0.34^a^	0.068
28d	4.03 ± 0.94	4.37 ± 1.02	4.99 ± 1.16	0.367
CSFV-Ab (ng/ml)				
0d	1.79 ± 0.02	1.18 ± 0.04	1.84 ± 0.04	0.577
14d	2.12 ± 0.16^b^	2.36 ± 0.19^ab^	2.80 ± 0.21^a^	0.058
28d	2.58 ± 0.15	2.64 ± 0.25	2.99 ± 0.21	0.329
IgA (μg/ml)				
0d	50.58 ± 0.99	54.57 ± 1.27	54.04 ± 1.57	0.093
14d	70.33 ± 2.17^b^	75.43 ± 3.16^ab^	78.51 ± 1.11^a^	0.069
28d	80.87 ± 5.57	85.17 ± 4.36	87.63 ± 4.03	0.599
IgG (mg/ml)				
0d	0.95 ± 0.04	0.86 ± 0.05	0.94 ± 0.07	0.421
14d	1.05 ± 0.07^b^	1.08 ± 0.07^ab^	1.28 ± 0.07^a^	0.067
28d	0.98 ± 0.07	1.21 ± 0.15	1.26 ± 0.11	0.198
IgM (mg/ml)				
0d	1.26 ± 0.04	1.35 ± 0.03	1.32 ± 0.05	0.335
14d	0.59 ± 0.05^b^	0.73 ± 0.05^ab^	0.77 ± 0.06^a^	0.074
28d	0.71 ± 0.07	0.83 ± 0.11	0.89 ± 0.06	0.315
IL-6 (pg/ml)	26.03 ± 0.15	25.42 ± 0.22	25.83 ± 0.20	0.109
TNF-α (pg./ml)	48.91 ± 2.95^a^	26.26 ± 3.38^b^	13.78 ± 1.73^c^	<0.01
IL-1β (pg./ml)	123.1 ± 9.03	102.81 ± 9.71	114.95 ± 7.28	0.269
IL-10 (pg./ml)	17.62 ± 2.56^b^	23.35 ± 3.09^ab^	27.15 ± 1.71^a^	0.052
IFN-γ (pg/mL)	7.46 ± 0.99	8.40 ± 1.02	9.74 ± 0.68	0.236

Values are represented as the mean and SEM. ^a–c^ Means within a row without a common superscript differ significantly (*p* < 0.05). PRV-Ab, swine pseudorabies antibody; CSFV-Ab, classical swine fever antibody; IgA, immunoglobulin A; IgG, immunoglobulin G; IgM, immunoglobulin M; TNF-α, tumor necrosis factor α; IL-1β, interleukin-1β; IFN-γ, interferon γ; IL-10, interleukin-10; IL-6, interleukin-6.

### 3.3 Effect of YAM on intestinal mucosal immunity of weaned piglets

**Figure 1:**
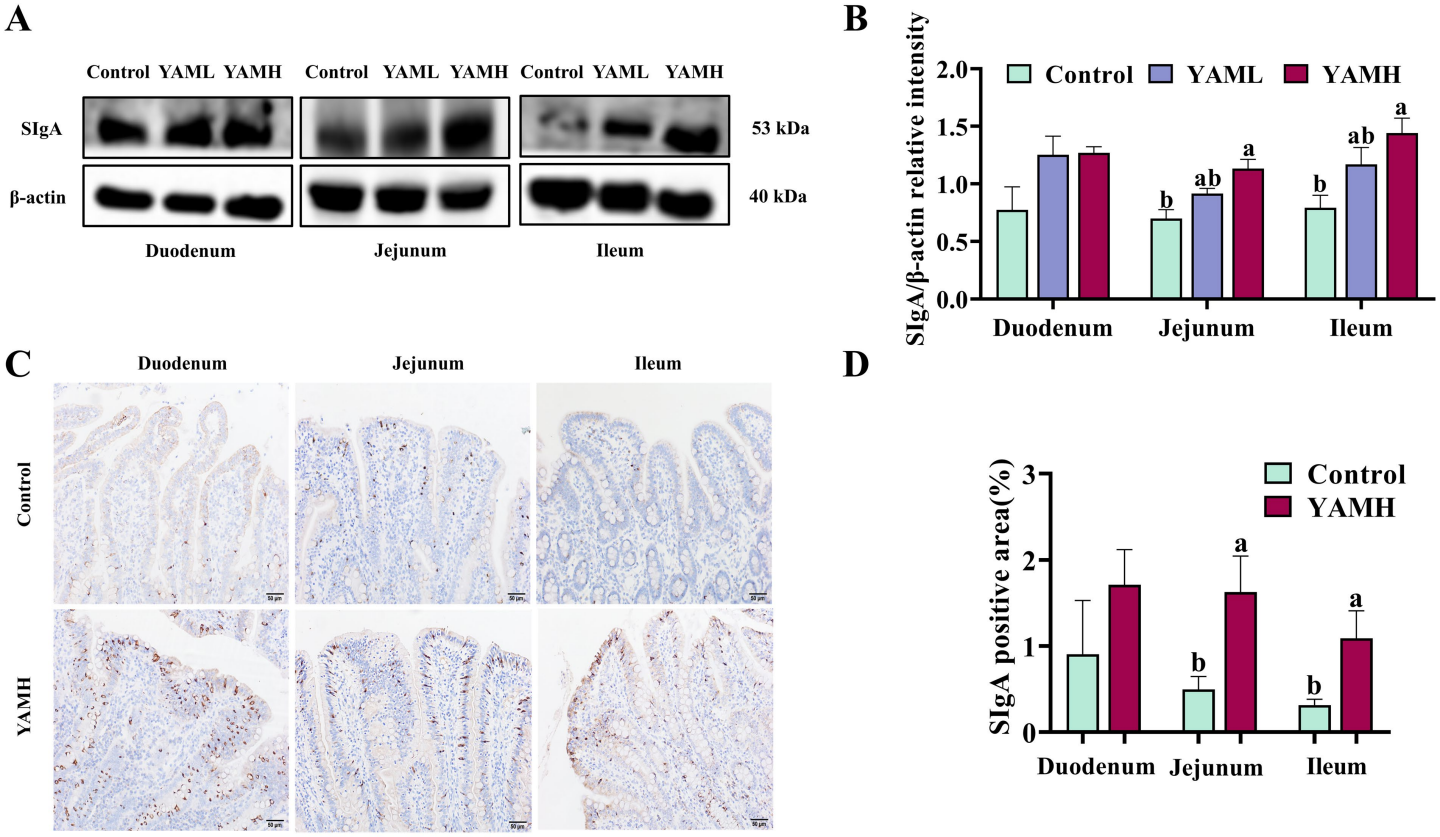
Effect of YAM on intestinal mucosal immunity of weaned piglets. **(A,B)**. Effect of YAM on intestinal SIgA protein expression level in weaned piglets; **(C,D)**. Immunohistochemical staining was used to detect the impact of YAM on the intestinal SIgA expression of weaned piglets (200 × scale bar 50 μm); SIgA, secretory immunoglobulin A.

The effects of dietary YAM addition on intestinal mucosal immunity in weaned piglets are shown in Figure 1. Compared with the control group, dietary YAM supplementation increased the protein expression of SIgA in the duodenum, jejunum and ileum of weaned piglets. This increase was particularly significant in the jejunum and ileum (*p* < 0.05, Figures 1A,B). Additionally, immunohistochemical analysis showed a significant increase in the protein expression of SIgA in the jejunum and ileum of weaned piglets in the YAMH group compared to the control group (*p* < 0.05, Figures 1C,D).

### 3.4 Effect of YAM on intestinal health of weaned piglets

The effect of YAM on the intestinal integrity of weaned piglets is shown in Table 6. In the duodenum, compared with the control group, the intestinal VH of weaned piglets significantly increased in both YAML and YAMH groups, with the YAMH group showing significant increase in the V/C ration (*p* < 0.05). In the jejunum, both test groups significantly increased V/C, and the YAMH group significantly reduced the CD of weaned piglets (*p* < 0.05). In the ileum, the test groups significantly increased the intestinal VH and V/C ratio, while the YAMH group significantly decreased the CD of weaned piglets (*p* < 0.05).

**Table 6:** Effect of YAM on intestinal morphology of weaned piglets.

Item	Control	YAML	YAMH	*p* value
Duodenum				
VH (cm)	0.27 ± 0.01^b^	0.31 ± 0.01^a^	0.32 ± 0.01^a^	0.019
CD (cm)	0.50 ± 0.02	0.47 ± 0.02	0.41 ± 0.03	0.104
V/C	0.55 ± 0.02^b^	0.68 ± 0.04^ab^	0.83 ± 0.07^a^	0.012
Jejunum				
VH (cm)	0.31 ± 0.01	0.34 ± 0.01	0.34 ± 0.01	0.068
CD (cm)	0.46 ± 0.02^a^	0.39 ± 0.01^ab^	0.38 ± 0.03^b^	0.059
V/C	0.70 ± 0.03^b^	0.88 ± 0.04 ^a^	0.94 ± 0.07^a^	0.030
Ileum				
VH (cm)	0.29 ± 0.01^b^	0.32 ± 0.00^a^	0.32 ± 0.01^a^	0.037
CD (cm)	0.46 ± 0.00^a^	0.42 ± 0.01^ab^	0.41 ± 0.02^b^	0.058
V/C	0.64 ± 0.01^b^	0.76 ± 0.03^a^	0.81 ± 0.03^a^	0.007

Values are represented as the mean and SEM. ^a–b^ Means within a row without a common superscript differ significantly (*p* < 0.05). Various serum biochemical parameters were measured, including ALB, albumin; ALT, alanine aminotransferase; ALP, serum alkaline phosphatase; GLU, glucose; TC, total cholesterol; TG, triglyceride; HDL-C, high-density lipoprotein cholesterol; LDL-C, low-density lipoprotein cholesterol; UREA, blood urea nitrogen.

**Figure 2:**
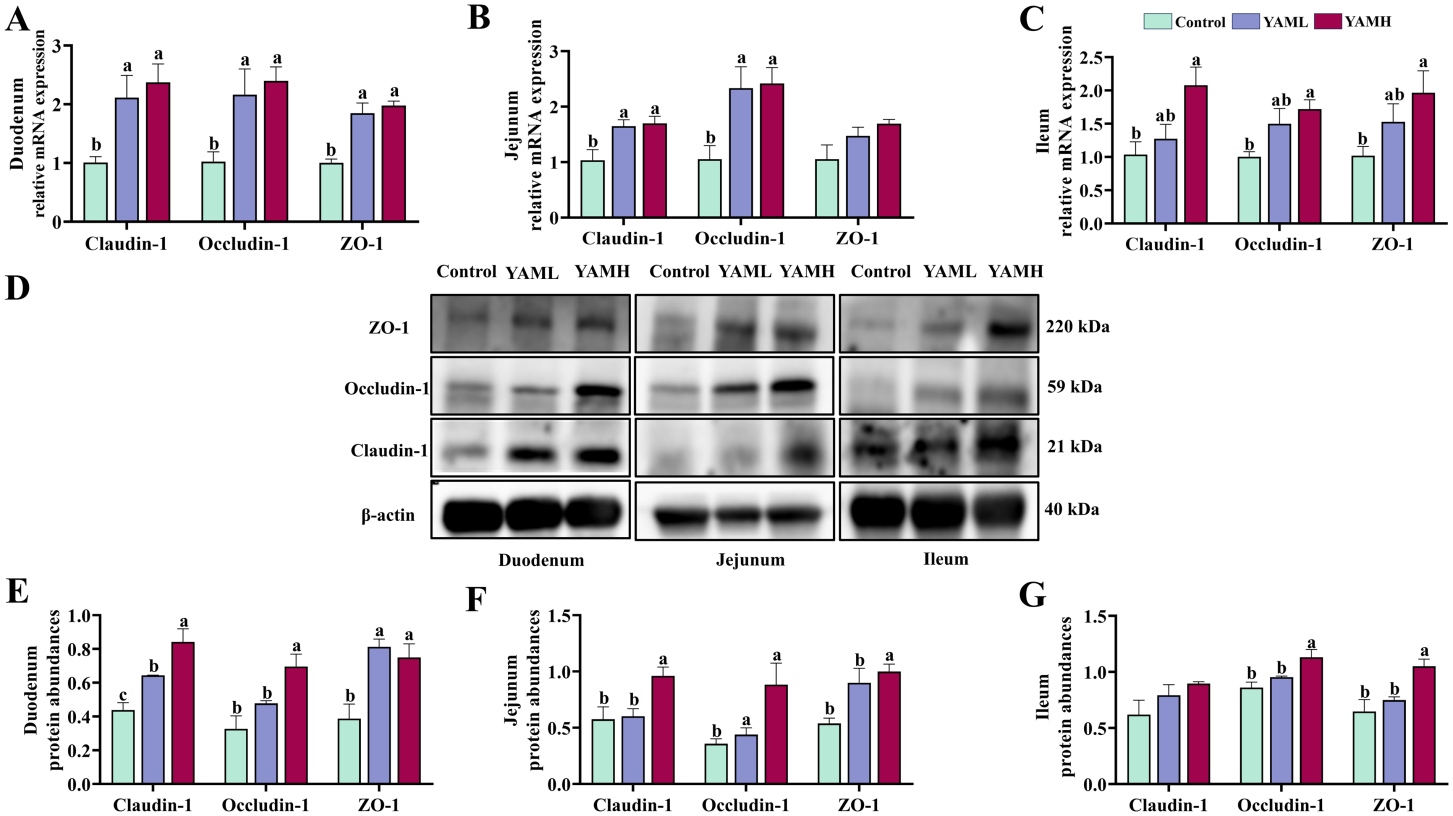
Effect of YAM on intestinal health of weaned piglets. **(A–C)** The mRNA expressions of *ZO-1, Claudin-1*, and *Occludin* in the duodenum, jejunum, and ileum; **(D)** the representative bands for the western blot of ZO-1, Claudin-1 and Occludin-1; **(E–G)** Protein expression levels of ZO-1/*β*-actin, Occludin-1 /β-actin, Claudin-1/β-actin in duodenum, jejunum and ileum.

The effect of YAM on the intestinal tight junctions (TJs) of weaned piglets is shown in Figure 2. After dietary supplementation with YAM, the gene expression levels of *Claudin-1, Occludin-1* and *ZO-1* were significantly higher in the duodenum of weaned piglets compared to the control group (*p* < 0.05, Figure 2A). In the jejunum, the YAM group significantly increased the expression levels of *Claudin-1* and *Occludin-1* (*p* < 0.05, Figure 2B). The YAMH group significantly increased the expression levels of ileum *Claudin-1, Occludin-1* and *ZO-1* (*p* < 0.05, Figure 2C). Furthermore, the protein levels of TJs were also detected (Figure 2D). Claudin-1 and ZO-1 protein expression levels were significantly higher in the duodenum in both the YAML and YAMH groups compared to the control group (*p* < 0.05, Figure 2E). In the jejunum, Occludin-1 protein expression levels were significantly higher in the YAML group, and Claudin-1, Occludin-1 and ZO-1 protein expression levels were significantly higher in the YAMH group (*p* < 0.05, Figure 2F). Similarly, YAMH supplementation significantly elevated Occludin-1 and ZO-1 protein expression levels in the ileum (*p* < 0.05, Figure 2G).

### 3.5 Effect of YAM on the composition of the colonic microbial community of weaned piglets

**Figure 3:**
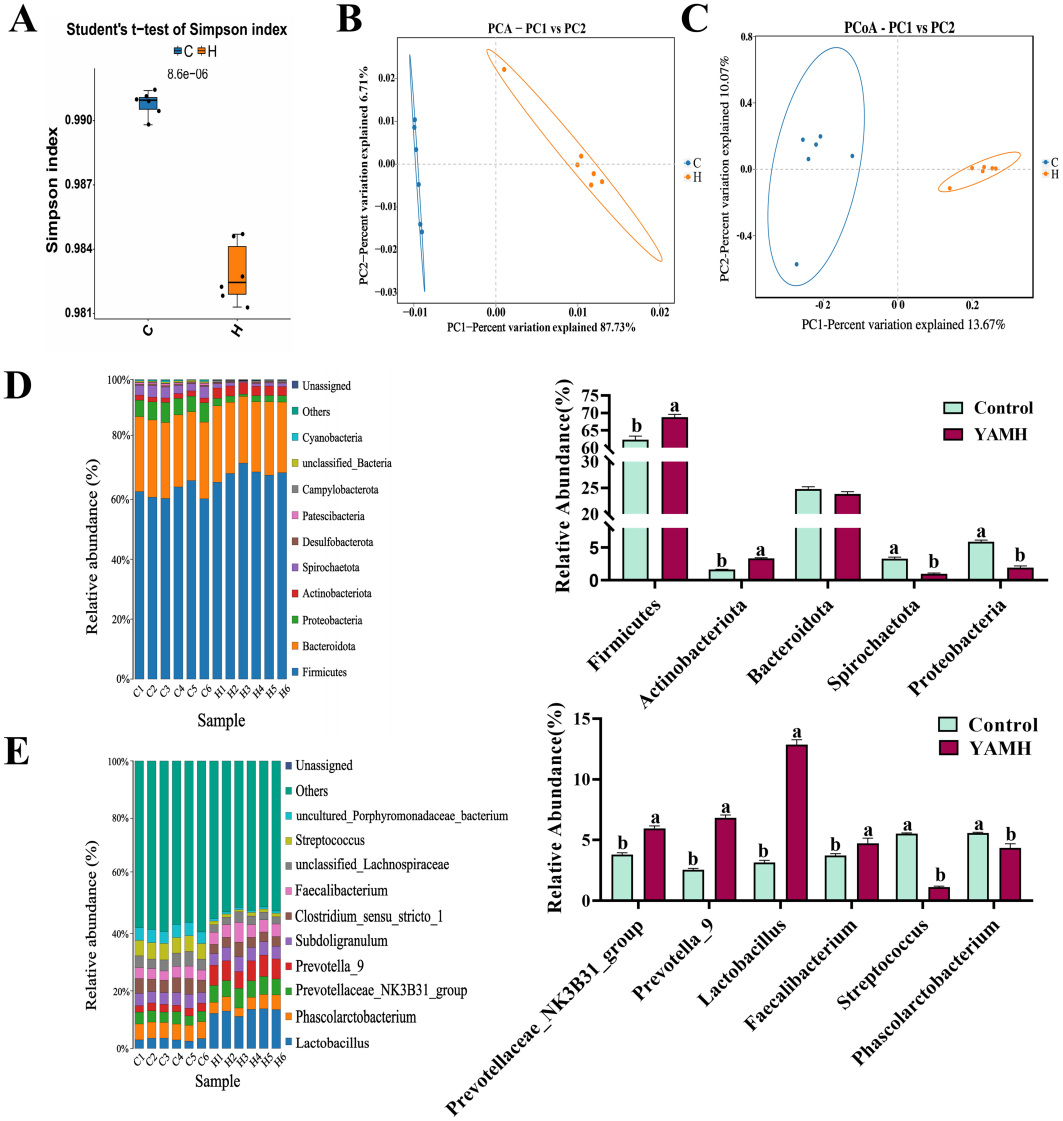
Effect of YAM on colonic flora microbiology in weaned piglets. **(A)** Simpson; **(B)** PCA chart, principal component analysis; **(C)** PCoA Chart, principal coordinate analysis; **(D)** Effect of YAM on phylum level flora; **(E)** Effect of YAM on genus-level flora.

We analyzed the intestinal microbiota through high-throughput sequencing of 16Sr RNA amplicons. Simpson’s index significantly decreased in the YAMH group compared to the control group (*p* < 0.05, Figure 3A). PCA and PCoA plots revealed that the distances between the two groups were significantly apart, while the distances within the groups were similar (Figures 3B,C). At the phylum level, the YAMH group showed a significant increase in the abundance of the dominant bacterial groups such as *Firmicutes* and *Actinobacteria* compared to the control group. In contrast, the abundance of the harmful bacteria such as *Spirochaetato* and *Proteobacteria*, was significantly decreased in the YAMH group (*p* < 0.05, Figure 3D). At the genus level, the YAMH group exhibited a significant increase in the abundance of the beneficial bacterial groups, including *Prevotellaceae_NK3B31*_*group*, *Prevotella-9, Lactobacillus*, and *Faecalibacterium.* Furthermore, the YAM treatment group significantly reduced the abundance of the harmful bacteria, such as *Streptococcus* and *Phascolarctobacterium* (*p* <0.05, Figure 3E). These results suggest that the addition of YAM to the diet can alter the diversity and structure of intestinal microbiota in piglets.

### 3.6 Correlation analysis

**Figure 4:**
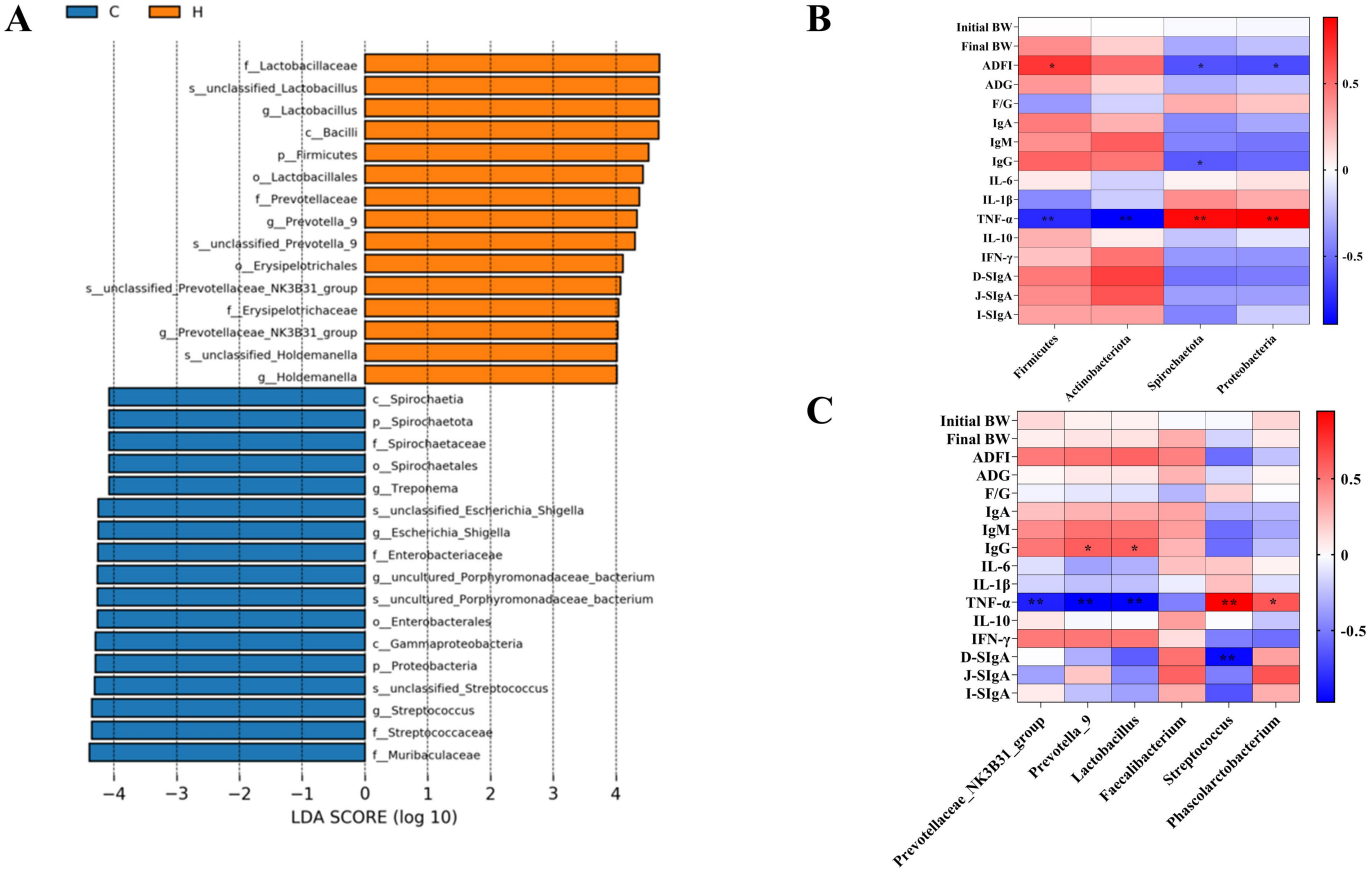
Correlation analysis. **(A)** LEfSe analysis identified the phylum to a genus whose abundances significantly differed in each group; **(B)** Correlation analysis of gate level, growth performance and immune markers **(C)** Correlation analysis of genus level, growth performance and immune indicators.

Correlation analysis was conducted to evaluate the relationships between piglet growth performance, immune factors, and gut microbiota, as shown in Figure 4. LEfSe multi-stage species differential discrimination analysis revealed that *f-lactobacillaceae*, *S-unclassified-lactobacillus* and *g-lactobacillus* were the most abundant in the YAMH group (Figure 4A). At the phylum level, *Firmicutes* were positively correlated with ADFI and negatively correlated with TNF-*α* (*p* < 0.05). *Actinobacteria* were negatively correlated with TNF-α (*p* < 0.05). *Spirochaetato* were negatively correlated with ADFI and IgG and positively correlated with TNF-α (*p* < 0.05). Similarly, *Proteobacteria* were negatively correlated with ADFI and positively correlated with TNF-α (*p* < 0.05, Figure 4B). At the genus level, IgG was positively correlated with *Prevotella-9* and *Lactobacillus* (*p* < 0.05), while TNF-α was negatively correlated with *Prevotellaceae_NK3B31_group*, *Prevotella-9* and *Lactobacillus* (*p* < 0.05). TNF-α was also positively correlated with *Streptococcus* and *Phascolarctobacterium,* D-SIgA was negatively correlated with *Streptococcus* (*p* < 0.05, Figure 4C).

## 4 Discussion

Weaning stress syndrome in piglets is a key factor affecting the economic efficiency of the pig industry (27–29). In the current context of promoting antibiotic-free feed, it is particularly important to develop green and safe feed additives derived from natural products to improve the intestinal health of early weaned piglets. The current scope of use of algae, plant extracts or traditional Chinese medicines, which are widely used in chicken, pig, cattle and aquaculture to improve animal growth performance, enhance immune function and regulate intestinal health. Dietary supplementation with astaxanthin improves alleviates carbohydrate diet-induced endoplasmic reticulum stress, immunosuppression and improves hepatic glucose metabolism in *C. argus* through activation of the AMP-activated protein kinase (AMPK) autophagy pathway (30); addition of lycium polysaccharides to piglet diets improves growth performance, antioxidant capacity, and immunity of weaned piglets, as well as reduces the incidence of diarrhea and regulates the composition of the intestinal microflora (31); and dietary addition of fermented herbs can improve the intestinal structure and morphology of the jejunum of weaned piglets by increasing the height of jejunal villi, digestive enzyme activity, and regulating the intestinal bacterial flora to promote intestinal health and improve the growth performance of weaned piglets (32). As a medicinal and food substance, YAM is rich in polysaccharides, phenolics, flavonoids, amino acids, saponins, urea sacs, mucins, and other active ingredients, which have immunomodulatory, antioxidant, anti-inflammatory, and antidigestive pharmacological effects (18). Some studies have shown that the polysaccharides (33), proteins (34), and diosgenin (35) of YAM are able to significantly improve animal health by regulating the intestinal flora, enhancing the intestinal barrier function, and inhibiting the proliferation of pathogens. At present, there have been no reports on the growth-regulating effect of YAM on piglets. This study is based on the potential pharmacological activity of YAM as a feed additive for weaned piglets, aiming to alleviate weaning stress syndrome in piglets and further explore its mechanism of action.

The stress experienced during weaning can lead to several issues in piglets, including diarrhea, impaired immune function, and stunted growth (27). The addition of YAM to piglet diets, however, resulted in improved growth outcomes. Specifically, we observed an increased in final body weight and average daily gain, along with a reduction in the feed-to-weight ratio and a decrease in the rate of diarrhea among piglets. Digestibility is a key measure of how effectively animals process and absorb nutrients from feed. However, as the digestive system of weaned piglets has not yet fully developed, there is insufficient secretion of gastric acid and digestive enzymes, which in turn affects the digestion and absorption of nutrients and ultimately hinders growth (36). In our study, the inclusion of YAM in the diet significantly improved the digestibility of crude protein and crude fat in weaned piglets, contributing to improved growth performance.

Oxidative stress plays an important role in diseases such as hepatitis (37). Studies have shown that hepatitis can lead to an increase in intracellular free radicals and oxidative substances, which in turn lead to oxidative stress. The production of oxidative stress will lead to aggravation of oxidative damage to liver cells and accelerate the further development of liver disease (38). ALT is mainly found in liver cells, and elevated ALT levels in the blood are usually associated with liver damage (39). It is not only closely related to oxidative stress and inflammatory response, but also affects growth performance by inhibiting nutrient metabolism and energy supply. In our study, the addition of yam reduced serum ALT activity in piglets. Based on the pharmacological activities of yam, such as antioxidant and anti-inflammatory effects (33, 40), it is speculated that yam may reduce cellular damage by alleviating oxidative stress and inflammatory damage, thereby reducing the release of ALT in the blood. TC and LDL-C are markers of lipid uptake and metabolism in the body (41). It is worth noting that the TC and LDL-C levels in the serum of the yam-supplemented group were significantly lower, indicating that yam may have a positive effect on lipid metabolism. In summary, our results show that adding yam to the diet can improve the digestibility of nutrients in piglets and regulate biochemical enzyme activity, thereby benefiting the healthy growth of piglets.

Antibody titers, which reflect the ability of a vaccine to generate specific immunity, are closely linked to an animal’s overall health. PRV and CSFV are both highly prevalent diseases in nursery pigs, and their antibody levels serve as important markers of immune function (42). Our results showed that PRV-Ab and CSFV-Ab levels were significantly higher in the blood of piglets in the experimental group compared to the control group, indicating that YAM can promote the disease resistance of piglets. The immune response of animals to infection is closely related to immunoglobulin levels. IgG, IgM and IgA are the main immunoglobulins that protect animals from infection (43). The Fc region of IgG interacts with Fc*γ*R and FcRn to provide pro- or anti-inflammatory effects depending on the specific situation. IgM exhibits its anti-inflammatory activity through IgM anti-leukocyte autoantibodies (IgM-ALA). IgA has the function of inhibiting the adhesion of bacteria and viruses to epithelial cells and neutralizing intracellular and extracellular viral and bacterial toxins. It is considered to be an important first line of defense against many invading pathogens (37). Our study found that YAM supplementation increased the serum IgA, IgG and IgM levels of piglets, indicating that YAM has an immune-enhancing effect, and this enhancement was particularly pronounced on day 14. IL-10 is a key anti-inflammatory mediator that protects the host from excessive immune responses to pathogens and the microbiota, while also reducing immune cell activation and cytokine production in innate immune cell populations (44). IFN-*γ* is involved in both acquired and innate immune responses in the body, has antiviral infection functions, and plays a key role in host defense (44). Stimulation of piglets with weaning stress or pathogen infection leads to increased expression and secretion of pro-inflammatory factors IL-6, TNF-*α* and IL-1β (3). In this study, YAM significantly reduced the protein expression level of TNF-α in the serum, while significantly increasing the expression levels of IL-10 and IFN-γ. Therefore, the results of this study show that YAM supplementation can enhance the immune function and disease resistance of piglets by increasing the expression level of immunoglobulins and antibody titers in piglet blood and reducing the level of pro-inflammatory cytokines. In addition, glucocorticoid receptors (GR) may also be involved in regulating the NF-kB signaling pathway, reducing the production of pro-inflammatory factors TNF-a and IL-6, and thereby reducing immune stress in lymphocytes (45).

As an important component of the intestinal immune barrier, SIgA plays an important role in maintaining intestinal health by clearing pathogenic microorganisms and interacting with intestinal symbiotic microorganisms (5, 46). Therefore, the secretion of sufficient SIgA in the intestine is essential for intestinal homeostasis. In recent years, multiple studies have further confirmed the positive correlation between SIgA and growth performance. SIgA deficiency may lead to damage to the intestinal mucosa, thereby reducing digestion and absorption, resulting in weight loss and affecting growth and developmentt (47). However, weanling piglets are usually unable to secrete sufficient SIgA due to the forced interruption of maternal immunoglobulins and the underdeveloped intestinal immune system. WB and immunohistochemical results showed that the expression level of SIgA in the intestinal tract of piglets increased to varying degrees. Therefore, YAM enhances the immune function of the body by increasing the level of SIgA, which may in turn affect the growth performance of piglets.

The intestinal tract is the primary site for nutrition digestion and absorption, and its structural integrity is essential for the proper digestion and absorption in weaned piglets. Intestinal morphology, including villus height (VH), crypt depth (CD), and villus-to-crypt ratio (V/C), reflects the digestive and absorptive capabilities and overall intestinal health (48). After weaning, changes in intestinal epithelial morphology, such as atrophy of small intestinal villi, crypt hyperplasia, and reduced digestive enzyme activity, suggest impaired intestinal mucosal function and decreased digestive and absorptive efficiency (5). In this experiment, adding YAM to the diet of weaned piglets, significantly increased the height of intestinal villi, villous-to-crypt ratio, and reduced the depth of crypts. The intestinal mechanical barrier is the first line of defense against harmful substances entering the intestinal mucosa, and it plays a crucial role in maintaining intestinal homeostasis. The barrier is mainly composed of epithelial cells and TJs (Claudin-1, Occludin-1, and ZO-1), such as ZO-1, which are directly or indirectly anchored to the actin-based cytoskeleton, and then form a selective permeability barrier (49); Claudin-1 is involved in the formation of the structural basis of tight junctions, which modulate ion-selective inter-cellular permeability and enhance the barrier tightness (50); Occludin is the first intramembrane tight junction protein discovered, which is involved in the formation of tight junctions and helps to maintain the integrity and permeability of the intestinal barrier; at the same time, it plays the role of adhesion molecule between cells, and this adhesion not only helps to maintain the structural integrity of the tissues but also contributes to the signaling between the cells and the coordination of cell functions; in addition, it also It has a protective role in the intestinal tract, it can promote the renewal of intestinal morphology and intestinal barrier integrity, thus maintaining the normal physiological function of the intestinal tract, it can also form a fence to prevent the cells from spreading to the top and basolateral membranes, thus maintaining the polarity of the epithelial cells (51). Early weaning in piglets reduces the expression of intestinal TJs, resulting in intestinal barrier damage, increased permeability, the entry of harmful substances into the intestinal lumen, and microbiota dysbiosis. In our study, the addition of YAM to the diet increased the expression levels of intestinal *Claudin-1, Occludin-1*, and *ZO-1* genes and proteins, consistent with our previous findings showing that YAM improves intestinal morphological integrity in weaned piglets. These results indicate that YAM plays an active role in enhancing the integrity of the intestinal barrier and reducing intestinal permeability in weaned piglets. The appropriate amount of YAM supplementation was beneficial to the intestinal health of weaned piglets. Overall, the results showed that YAM supplementation improves gut health by improving gut morphology and barrier integrity.

Microorganisms colonized in the mammalian gut play an essential role in regulating intestinal digestion and absorption, maintaining the intestinal barrier, immune homeostasis, and other physiological processes (52). An imbalance in the intestinal microbiota can lead to apoptosis, intestinal barrier damage, and immune dysfunction, all of which affect the intestinal development and health of weaned piglets (53). At the phylum level, *Firmicutes* accelerate the metabolism of sugars and absorb a large amount of energy from a small amount of food while promoting fat accumulation in the piglet’s body and production of short-chain fatty acids. These fatty acids play an essential role in regulating immunity and maintaining normal physiological functions in the mammalian gastrointestinal tract. *Bacteroidota* promotes the fermentation of dietary fiber and oligosaccharides in the intestine to produce short-chain fatty acids, thereby enhancing intestinal activity, promoting the digestive function, and improving the intestinal environment. The ratio of *Firmicutes* and *Bacteroidota* is a key indicator of intestinal balance (54). In this study, the addition of YAM increased the abundance of *Firmicutes* and decreased the abundance of *Bacteroidetes* compared to the control group. The change in the ratio of these two phyla indicated that YAM supplementation could improve the intestinal digestion, absorption, and immune function of weaned piglets. *Actinobacteriota* is a beneficial bacterium that maintains the stability of the intestinal microbiota and strenghthens the immune system’s resistance (55). In this study, the YAM supplementation group showed a significant increase in the number of *Actinobacteriota* and a decrease in *Proteobacteria*, revealing that the intestinal flora of piglets after YAM supplementation has better stability and enhanced immunity. At the genus level, *Lactobacillus* promotes the production of secretory immunoglobulins, thereby increasing mucosal immunity and improving the intestinal barrier in weaned piglets (56). *Prevotella* is involved in the decomposition of starch, plant polysaccharides, and the catabolism of muscular mucin (57). *Faecalibacterium* produces butyrate, a short-chain fatty acid that plays a vital role in intestinal health and physiology. Butyrate is the primary energy source for colon cells to prevent inflammation and colon cancer. *Streptococcus* induces conditional pathogenic species such as *Streptococcus bovis* and *Streptococcus sanguis*, which are associated with sepsis and endocarditis, and their overgrowth is linked to piglet diarrhea. *Phascolarctobacterium* changes in diversity are associated with inflammatory bowel disease. In this study, adding YAM to the diet increased abundance of beneficial bacteria at the genus level, including *Prevotella*, *Faecalibacterium*, and *Lactobacillus*, while reducing harmful bacteria such as *Streptococcus* and *Phascolarctobacterium*. The results suggest that YAM supplementation improves the diversity of intestinal microbiota in weaned piglets. This, in turn, enhances immune defense, intestinal digestion and absorption, and overall intestinal health by regulating the gut microbiota.

## 5 Conclusion

Based on all the results, we demonstrated that the addition of YAM to piglet feed can alleviate piglet weaning stress syndrome by promoting growth performance, improving immune function and disease resistance, improving intestinal morphology and mucosal immunity, and regulating the intestinal microbial composition of piglets. These results highlight that YAM produces beneficial effects in the weaning transition of piglets. Notably, the addition of 2% YAM yielded the most favorable outcomes. These findings not only provide a new strategy to alleviate weaning stress syndrome in piglets but also promote the development and application of plant-derived feed additives. Nevertheless, there are still some shortcomings in this research. For example, which specific ingredients in YAM are responsible for these positive effects? This will be explored in more depth in future research.

## Data Availability

The datasets presented in this study can be found in online repositories. These data can be found in doi: 10.6084/m9.figshare.28705172.

## References

[1] Qiao L Dou X Song X Chang J Zeng X Zhu L. Replacing dietary sodium selenite with biogenic selenium nanoparticles improves the growth performance and gut health of early-weaned piglets. Anim Nutr. (2023) 15:99–113. doi: 10.1016/j.aninu.2023.08.003, PMID: 38023380 PMC10665811

[2] Zhang Y Wang Y Chen D Yu B Zheng P Mao X. Dietary Chlorogenic acid supplementation affects gut morphology, antioxidant capacity and intestinal selected bacterial populations in weaned piglets. Food Funct. (2018) 9:4968–78. doi: 10.1039/C8FO01126E, PMID: 30183786

[3] Li M Chen L Zhao Y Sun H Zhao L. Research on the mechanism of Hrp relieving Ipec-J2 cells immunological stress based on transcriptome sequencing analysis. Front Nutr. (2022) 9:944390. doi: 10.3389/fnut.2022.944390, PMID: 35911118 PMC9336541

[4] Zhao L Geng T Sun K Su S Zhao Y Bao N. Proteomic analysis reveals the molecular mechanism of *Hippophae Rhamnoides* polysaccharide intervention in Lps-induced inflammation of Ipec-J2 cells in piglets. Int J Biol Macromol. (2020) 164:3294–304. doi: 10.1016/j.ijbiomac.2020.08.235, PMID: 32888998

[5] Zhao L Yu J Liu Y Liu Y Zhao Y Li M-Y. The major roles of intestinal microbiota and Traf6/Nf-Κb signaling pathway in acute intestinal inflammation in mice, and the improvement effect by *Hippophae Rhamnoides* polysaccharide. Int J Biol Macromol. (2025) 296:139710. doi: 10.1016/j.ijbiomac.2025.139710, PMID: 39793780

[6] Wang S Guo C Zhou L Zhong Z Zhu W Huang Y. Effects of dietary supplementation with epidermal growth factor-expressing *Saccharomyces Cerevisiae* on duodenal development in weaned piglets. Br J Nutr. (2016) 115:1509–20. doi: 10.1017/S0007114516000738, PMID: 26983845

[7] Campbell JM Crenshaw JD Polo J. The biological stress of early weaned piglets. J Anim Sci Biotechnol. (2013) 4:19. doi: 10.1186/2049-1891-4-19, PMID: 23631414 PMC3651348

[8] Liu Y Azad MAK Ding S Zhu Q Blachier F Yu Z. Dietary bile acid supplementation in weaned piglets with intrauterine growth retardation improves colonic microbiota, metabolic activity, and epithelial function. J Anim Sci Biotechnol. (2023) 14:99. doi: 10.1186/s40104-023-00897-2, PMID: 37438768 PMC10339644

[9] Liao SF Nyachoti M. Using probiotics to improve swine gut health and nutrient utilization. Anim Nutr. (2017) 3:331–43. doi: 10.1016/j.aninu.2017.06.007, PMID: 29767089 PMC5941265

[10] Ma J Long S Wang J Gao J Piao X. Microencapsulated essential oils combined with organic acids improves immune antioxidant capacity and intestinal barrier function as well as modulates the hindgut microbial Community in Piglets. J Anim Sci. Biotechnol. (2022) 13:16. doi: 10.1186/s40104-021-00670-3, PMID: 35144681 PMC8832826

[11] Huang X Jiang F Chen X Xian Y. Plant-derived polysaccharides benefit weaned piglets by regulating intestinal microbiota: a review. J Agric Food Chem. (2024) 72:28225–45. doi: 10.1021/acs.jafc.4c08816, PMID: 39663725

[12] Tucker BS Craig JR Morrison RS Smits RJ Kirkwood RN. Piglet viability: a review of identification and pre-weaning management strategies. Animals. (2021) 11:2902. doi: 10.3390/ani11102902, PMID: 34679923 PMC8532860

[13] Yu Z Li M. Application of nutrition interventions strategy to enhance fish flesh quality. J Food Compos Anal. (2024) 138:107010. doi: 10.1016/j.jfca.2024.107010, PMID: 40128009

[14] Yu Z Zhao L Zhao J-L Xu W Guo Z Zhang A-Z. Dietary Taraxacum Mongolicum polysaccharide ameliorates the growth, immune response, and antioxidant status in association with Nf-Κb, Nrf2 and Tor in Jian carp (*Cyprinus Carpio* Var. Jian). Aquaculture. (2022) 547:737522. doi: 10.1016/j.aquaculture.2021.737522

[15] Du J-H Xu M-Y Wang Y Lei Z Yu Z Li M-Y. Evaluation of Taraxacum Mongolicum flavonoids in diets for *Channa Argus* based on growth performance, immune responses, apoptosis and antioxidant defense system under lipopolysaccharide stress. Fish Shellfish Immunol. (2022) 131:1224–33. doi: 10.1016/j.fsi.2022.11.034, PMID: 36414130

[16] Czech A Klimiuk K Sembratowicz I. The effect of thyme herb in diets for fattening pigs on their growth performance and health. PLoS One. (2023) 18:e0291054. doi: 10.1371/journal.pone.0291054, PMID: 37796995 PMC10553308

[17] Maass N Bauer J Paulicks B Böhmer B Roth-Maier D. Efficiency of *Echinacea Purpurea* on performance and immune status in pigs. J Anim Physiol Anim Nutr. (2005) 89:244–52. doi: 10.1111/j.1439-0396.2005.00501.x, PMID: 15972074

[18] Zhang L Wang S Zhang W Chang G Guo L Li X. Prospects of yam (Dioscorea) polysaccharides: structural features, bioactivities and applications. Food Chem. (2024) 446:138897. doi: 10.1016/j.foodchem.2024.138897, PMID: 38430768

[19] Huang R Shen M Yu Y Liu X Xie J. Physicochemical characterization and immunomodulatory activity of sulfated Chinese yam polysaccharide. Int J Biol Macromol. (2020) 165:635–44. doi: 10.1016/j.ijbiomac.2020.09.213, PMID: 33010270

[20] Li Z Xiao W Xie J Chen Y Yu Q Zhang W. Isolation, characterization and antioxidant activity of yam polysaccharides. Food Secur. (2022) 11:800. doi: 10.3390/foods11060800, PMID: 35327223 PMC8954450

[21] Zhou S Huang G Chen G. Extraction, structural analysis, derivatization and antioxidant activity of polysaccharide from Chinese yam. Food Chem. (2021) 361:130089. doi: 10.1016/j.foodchem.2021.130089 34029907

[22] Guo L Chang Y Sun Z Deng J Jin Y Shi M. Effects of Chinese yam polysaccharide on intramuscular fat and fatty acid composition in breast and thigh muscles of broilers. Food Secur. (2023) 12:1479. doi: 10.3390/foods12071479, PMID: 37048300 PMC10094610

[23] Zhang J Jin Y Cao M Deng J Chang Y Shi M. Effects of dietary Chinese yam polysaccharide copper complex on growth performance, immunity, and antioxidant capacity of broilers. Front Vet Sci. (2023) 10:1123002. doi: 10.3389/fvets.2023.1123002 36875994 PMC9978188

[24] Fu L Sun M Dong W Zhang G Han D Zang J. Effects of compound of hawthorn (*Crataegus Pinnatifida*) and Chinese yam (Dioscorea Opposita Thunb.) extracts on growth performance, intestinal health, and immune function in weaned pigs. Anim Sci J. (2022) 93:e13790. doi: 10.1111/asj.13790, PMID: 36504192

[25] Tiwari UP Mandal RK Neupane KR Mishra B Jha R. Starchy and fibrous feedstuffs differ in their in vitro digestibility and fermentation characteristics and differently modulate gut microbiota of swine. J Anim Sci Biotechnol. (2022) 13:53. doi: 10.1186/s40104-022-00699-y, PMID: 35501888 PMC9063073

[26] Huang Q Li W Jing X Liu C Ahmad S Huang L. Naringin’s alleviation of the inflammatory response caused by *Actinobacillus Pleuropneumoniae* by downregulating the Nf-Κb/Nlrp3 Signalling pathway. Int J Mol Sci. (2024) 25:1027. doi: 10.3390/ijms25021027, PMID: 38256101 PMC10816821

[27] Tang X Xiong K Fang R Li M. Weaning stress and intestinal health of piglets: a review. Front Immunol. (2022) 13:1042778. doi: 10.3389/fimmu.2022.1042778, PMID: 36505434 PMC9730250

[28] Upadhaya S-D Kim I-H. The impact of weaning stress on gut health and the mechanistic aspects of several feed additives contributing to improved gut health function in weanling piglets—a review. Animals. (2021) 11:2418. doi: 10.3390/ani11082418 34438875 PMC8388735

[29] Yu L Li H Peng Z Ge Y Liu J Wang T. Early weaning affects liver antioxidant function in piglets. Animals. (2021) 11:2679. doi: 10.3390/ani11092679, PMID: 34573645 PMC8469846

[30] Li M-Y Liu Y-Z Chen X-M Niu X-T Chen L Zhao L. Astaxanthin ameliorates high-carbohydrate diet-induced Er stress, immunosuppression and hepatic glucose metabolism through Ampk/autophagy pathway in *Channa Argus*. Aquaculture. (2025) 598:742010. doi: 10.1016/j.aquaculture.2024.742010

[31] Yin Y Wang F Yang M Tan B Yin Y Chen J. *Lycium Barbarum* polysaccharides as antibiotic substitutes improve growth performance, serum immunity, antioxidant status, and intestinal health for weaned piglets. Front Microbiol. (2022) 12:819993. doi: 10.3389/fmicb.2021.819993, PMID: 35281314 PMC8914510

[32] Chen G Li Z Liu S Tang T Chen Q Yan Z. Fermented Chinese herbal medicine promoted growth performance, intestinal health, and regulated bacterial microbiota of weaned piglets. Animals. (2023) 13:476. doi: 10.3390/ani13030476 36766365 PMC9913397

[33] Lu J Gong Y Gao Y Yang Y Zhang Y Zhang Z. Wolfberry, yam, and Chrysanthemum polysaccharides increased intestinal *Akkermansia Muciniphila* abundance and hepatic Yap1 expression to alleviate Dili. FASEB J. (2023) 37:e23286. doi: 10.1096/fj.202301388R, PMID: 37950623

[34] Lu J Qin H Liang L Fang J Hao K Song Y. Yam protein ameliorates cyclophosphamide-induced intestinal immunosuppression by regulating gut microbiota and its metabolites. Int J Biol Macromol. (2024) 279:135415. doi: 10.1016/j.ijbiomac.2024.135415, PMID: 39245119

[35] He Y Luo R Xia M Liu J Yao Y Min F. Orally administered Diosgenin alleviates colitis in mice induced by dextran sulfate sodium through gut microbiota modulation and short-chain fatty acid generation. J Med Food. (2022) 25:261–71. doi: 10.1089/jmf.2021.K.0086, PMID: 35320010

[36] Wang C Zhong Y Liu H Wang H Li Y Wang Q. Effects of dietary supplementation with tea residue on growth performance, digestibility, and diarrhea in piglets. Animals. (2024) 14:584. doi: 10.3390/ani14040584 38396552 PMC10886095

[37] Bruzeau C Cook-Moreau J Pinaud E Le Noir S. Contribution of immunoglobulin enhancers to B cell nuclear organization. Front Immunol. (2022) 13:877930. doi: 10.3389/fimmu.2022.877930, PMID: 35812441 PMC9263370

[38] Zhang X Wu X Hu Q Wu J Wang G Hong Z. Mitochondrial DNA in liver inflammation and oxidative stress. Life Sci. (2019) 236:116464. doi: 10.1016/j.lfs.2019.05.020 31078546

[39] Shi Y-C Zhao Y-R Zhang A-Z Zhao L Yu Z Li M-Y. Hexavalent chromium-induced toxic effects on the hematology, redox state, and apoptosis in *Cyprinus Carpio*. Reg Stud Mar Sci. (2022) 56:102676. doi: 10.1016/j.rsma.2022.102676

[40] Park S-Y Truong V-L Jeon S-G Choe S-Y Rarison RH Yoon B-H. Anti-inflammatory and prebiotic potential of ethanol extracts and mucilage polysaccharides from Korean yams (Dioscorea Polystachya and *Dioscorea Bulbifera*). Food Secur. (2025) 14:173. doi: 10.3390/foods14020173, PMID: 39856842 PMC11764955

[41] Yin S You T Tang J Wang L Jia G Liu G. Dietary licorice flavonoids powder improves serum antioxidant capacity and immune organ inflammatory responses in weaned piglets. Front Vet Sci. (2022) 9:942253. doi: 10.3389/fvets.2022.942253, PMID: 35958301 PMC9360566

[42] Brodin P. Immune-microbe interactions early in life: a determinant of health and disease Long term. Science. (2022) 376:945–50. doi: 10.1126/science.abk2189, PMID: 35617387

[43] Megha K Mohanan P. Role of immunoglobulin and antibodies in disease management. Int J Biol Macromol. (2021) 169:28–38. doi: 10.1016/j.ijbiomac.2020.12.073, PMID: 33340621

[44] Mu S Chen L Dong H Li S Zhang Y Yin S. Enhanced antigen-specific Cd8 T cells contribute to early protection against Fmdv through swine dc vaccination. J Virol. (2024) 98:e02002–23. doi: 10.1128/jvi.02002-23, PMID: 38289108 PMC10878267

[45] Niu X-T Sun C Zhao L Chen X-M Wang G-Q Li M-Y. The major role of glucocorticoid receptor (gr) in Astaxanthin alleviates immune stress in *Channa Argus* lymphocyte. Aquaculture. (2024) 584:740637. doi: 10.1016/j.aquaculture.2024.740637

[46] Zhao Y Wang J Wang H Huang Y Qi M Liao S. Effects of Gaba supplementation on intestinal Siga secretion and gut microbiota in the healthy and Etec-infected weanling piglets. Mediat Inflamm. (2020) 2020:1–17. doi: 10.1155/2020/7368483, PMID: 32565729 PMC7271228

[47] He X Lin Y Lian S Sun D Guo D Wang J. Selenium deficiency in chickens induces intestinal mucosal injury by affecting the mucosa morphology, Siga secretion, and Gsh-Px activity. Biol Trace Elem Res. (2020) 197:660–6. doi: 10.1007/s12011-019-02017-6, PMID: 31925740

[48] Tang X Xiong K. Intrauterine growth retardation affects intestinal health of suckling piglets via altering intestinal antioxidant capacity, glucose uptake, tight junction, and immune responses. Oxidative Med Cell Longev. (2022) 2022:2644205. doi: 10.1155/2022/2644205 PMC8957421 35345830

[49] Chen J Yu B Chen D Huang Z Mao X Zheng P. Chlorogenic acid improves intestinal barrier functions by suppressing mucosa inflammation and improving antioxidant capacity in weaned pigs. J Nutr Biochem. (2018) 59:84–92. doi: 10.1016/j.jnutbio.2018.06.005, PMID: 29986311

[50] Xu J Song J Zhang Y Wang Y Yang L Sha Y. Jinzhi protects lipopolysaccharide-treated mice against mortality by repairing intestinal mucosal barrier damage and intestinal microecology. Biomed Pharmacother. (2020) 123:109749. doi: 10.1016/j.biopha.2019.109749, PMID: 31846840

[51] Chelakkot C Ghim J Ryu SH. Mechanisms regulating intestinal barrier integrity and its pathological implications. Exp Mol Med. (2018) 50:1–9. doi: 10.1038/s12276-018-0126-x, PMID: 30115904 PMC6095905

[52] Tang X Liu X Zhong J Fang R. Potential application of *Lonicera Japonica* extracts in animal production: from the perspective of intestinal health. Front Microbiol. (2021) 12:719877. doi: 10.3389/fmicb.2021.719877, PMID: 34434181 PMC8381474

[53] Beaumont M Paës C Mussard E Knudsen C Cauquil L Aymard P. Gut microbiota derived metabolites contribute to intestinal barrier maturation at the suckling-to-weaning transition. Gut Microbes. (2020) 11:1268–86. doi: 10.1080/19490976.2020.1747335, PMID: 32352849 PMC7524271

[54] Wu S Wang L Cui B Wen X Jiang Z Hu S. Effects of vitamin a on growth performance, antioxidants, gut inflammation, and microbes in weaned piglets. Antioxidants. (2023) 12:2049. doi: 10.3390/antiox12122049, PMID: 38136169 PMC10740560

[55] Binda C Lopetuso LR Rizzatti G Gibiino G Cennamo V Gasbarrini A. Actinobacteria: a relevant minority for the maintenance of gut homeostasis. Dig Liver Dis. (2018) 50:421–8. doi: 10.1016/j.dld.2018.02.012, PMID: 29567414

[56] Bai M Liu H Wang S Shu Q Xu K Zhou J. Dietary Moutan cortex Radicis improves serum antioxidant capacity and intestinal immunity and alters colonic microbiota in weaned piglets. Front Nutr. (2021) 8:679129. doi: 10.3389/fnut.2021.679129, PMID: 34222303 PMC8247480

[57] Gao X Yu B Yu J Mao X Huang Z Luo Y. Developmental profiling of dietary carbohydrate digestion in piglets. Front Microbiol. (2022) 13:896660. doi: 10.3389/fmicb.2022.896660, PMID: 35572714 PMC9100932

